# HASP server: a database and structural visualization platform for comparative models of influenza A hemagglutinin proteins

**DOI:** 10.1186/1471-2105-14-197

**Published:** 2013-06-18

**Authors:** Xavier I Ambroggio, Jennifer Dommer, Vivek Gopalan, Eleca J Dunham, Jeffery K Taubenberger, Darrell E Hurt

**Affiliations:** 1 Bioinformatics and Computational Biosciences Branch, Office of Cyber Infrastructure and Computational Biology, National Institute of Allergy and Infectious Diseases, National Institutes of Health, Bethesda, MD, USA; 2 Viral Pathogenesis and Evolution Section, Laboratory of Infectious Diseases, National Institute of Allergy and Infectious Diseases, National Institutes of Health, Bethesda, MD, USA

**Keywords:** Influenza A, HASP, Hemagglutinin, Receptor binding, Membrane fusion, ROSETTA, Sialic acid, Flu, Homology modeling, Molecular visualization

## Abstract

**Background:**

Influenza A viruses possess RNA genomes that mutate frequently in response to immune pressures. The mutations in the hemagglutinin genes are particularly significant, as the hemagglutinin proteins mediate attachment and fusion to host cells, thereby influencing viral pathogenicity and species specificity. Large-scale influenza A genome sequencing efforts have been ongoing to understand past epidemics and pandemics and anticipate future outbreaks. Sequencing efforts thus far have generated nearly 9,000 distinct hemagglutinin amino acid sequences.

**Description:**

Comparative models for all publicly available influenza A hemagglutinin protein sequences (8,769 to date) were generated using the Rosetta modeling suite. The C-alpha root mean square deviations between a randomly chosen test set of models and their crystallographic templates were less than 2 Å, suggesting that the modeling protocols yielded high-quality results. The models were compiled into an online resource, the Hemagglutinin Structure Prediction (HASP) server. The HASP server was designed as a scientific tool for researchers to visualize hemagglutinin protein sequences of interest in a three-dimensional context. With a built-in molecular viewer, hemagglutinin models can be compared side-by-side and navigated by a corresponding sequence alignment. The models and alignments can be downloaded for offline use and further analysis.

**Conclusions:**

The modeling protocols used in the HASP server scale well for large amounts of sequences and will keep pace with expanded sequencing efforts. The conservative approach to modeling and the intuitive search and visualization interfaces allow researchers to quickly analyze hemagglutinin sequences of interest in the context of the most highly related experimental structures, and allow them to directly compare hemagglutinin sequences to each other simultaneously in their two- and three-dimensional contexts. The models and methodology have shown utility in current research efforts and the ongoing aim of the HASP server is to continue to accelerate influenza A research and have a positive impact on global public health.

## Background

Influenza A viruses (IAV) are among the most common causes of human respiratory infections and among the most significant because they cause high morbidity and mortality, both in annual epidemics and in unpredictable pandemics [1]. IAVs are enveloped negative-strand RNA viruses with segmented genomes containing 8 gene segments encoding at least 11 open reading frames [2]. IAVs are covered with proteins, notably hemagglutinin (HA) and neuraminidase (NA). The combination of alleles of HA and NA define strain nomenclature because of their variability and importance in pathogenicity, in host immunity, and in host species specificity. Currently, there are 17 subtypes of HA and 10 subtypes of NA.

HA is a glycosylated type I integral membrane protein that functions both as the viral receptor-binding protein and fusion protein. HA recognizes sialic-acid (SA) bound glycans with variable specificity for SA with α2-3 or α2-6 glycosidic linkages; these linkages are critical in determining host species specificity [3]. The HA protein is a trimer, with each monomer composed of a heavy (~40 kDa) and light (~20 kDa) chain cleaved from a single precursor. Given the HA diversity of IAVs, structural predictions are an important tool to map receptor-binding and antigenic regions.

To date, nearly 9,000 distinct HA alleles have been sequenced [4], which is roughly three orders of magnitude larger than the number of experimentally determined structures (Table 1). Given the large scale of HA sequencing efforts and the experimental barriers in determining HA structures, the gap between the experimental characterization of primary and tertiary structures is likely to persist. Despite the many changes HAs accumulate in their primary amino acid sequences as a result of antigenic shift and drift, the tertiary structures of even highly divergent HAs are largely conserved. For example, the HA structures of a group 1 H9 subtype (PDB ID: 1JSD [5]) and a group 2 H7 subtype (PDB ID: 1TI8 [6]) can be superposed with a C-alpha Root Mean Square Deviation (RMSD)of 2.8 Å over 467 amino acids, despite only 40% sequence identity. With this structural similarity between divergent HAs, comparative models of HA proteins may be of reasonable quality and hence useful in filling the information gap between HA sequence and structure space. Here we present the Hemagglutinin Structure Prediction (HASP) server, a database of HA comparative models of all publicly available HA sequences. The database is provided with an integrated molecular viewer to accelerate model analysis and hypothesis generation.

**Table 1:** HA crystal structures and templates selected for HASP server

**Subtype**	**PDB ID**^**a**^
H1	1RD8, 1RU7, 1RUY, 1RUZ, **1RV0**, 1RVT, **1RVX**, 1RVZ, 2WRG, 2WRH, **3GBN**, 3HTO, 3HTP, 3HTQ, **3HTT**, 3LZF, **3LZG**, 3M6S
H2	2WR7, 2WRB, 2WRC, 2WRD, 2WRE, 2WRF, **3KU3**, 3KU5, 3KU6
H3	1EO8, 1HA0, 1HGD, 1HGE, 1HGF, 1HGG, 1HGH, 1HGI, 1HGJ, 1HTM, 1KEN, 1MQL, **1MQM**, 1MQN, 1QFU, 1QU1, 2HMG, 2OJE, 2VIR, 2VIS, 2VIT, **2VIU**, 3EYM, 3HMG, 4HMG, 5HMG
H5	**1JSM**, 1JSN, 1JSO, 2FK0, 2IBX, 3FKU, **3GBM**, 3MGO
H7	**1TI8**
H9	**1JSD**, 1JSH, 1JSI

^a^PDB IDs in bold were selected as template structures for comparative modeling. The structures and the corresponding publications can be found online in the Research Collaboratory for Structural Biosciences PDB: http://www.rcsb.org/pdb.

## Construction and content

### Template preparation

All crystal structures of influenza HAs were retrieved from the Protein Data Bank (PDB) and filtered through the PISCES server [7] using a 95% sequence identity cutoff. Additional criteria included having an R-factor below 0.30 and a resolution better than 4.0 Å, resulting in a set of 12 structures (Table 1). Each of these structures was processed with the Molprobity server [8] to optimize hydrogen positions and rotamers for asparagine, glutamine, and histidine residues. A symmetric trimer for each model was then generated from the first (or only) monomer in the model by applying the crystallographic or non-crystallographic symmetry operators using the symmetry functionality of ROSETTA3, v3.2 [9]. The resulting models and their amino acid sequences were used for comparative modeling.

### Sequence alignments

All unique HA sequences were downloaded from the Influenza Research Database (8,769 as of June 2011) [10]. The template sequences were searched with each sequence using BLAST [11]. The top scoring high-scoring segment pair (hsp) was used as the alignment for comparative modeling.

### Model preparation

A conservative approach was taken in the modeling, where only amino acid types, side-chain conformations, and dihedrals were allowed to change, with insertions and deletions omitted from the model. This approach was chosen because of the high sequence similarity between the query and template HA sequences and the desire to retain as much information from the crystal structure as possible.

The models were created by mapping the query sequence onto the template sequence from the hsp alignment using the fixed-backbone design functionality of ROSETTA3, v3.2 [9]. Rotamers with χ_1_ angles ±1 standard deviation from canonical Dunbrack rotamers were included in modeling. Energy minimization of the side chain dihedral angles was also performed. For positions with identities between the query and template sequences in the hsp alignments, the side-chain conformation of the template structure was retained. Only aligned residues in the hsp alignment are present in the final models.

### Model quality estimates

**Figure 1:**
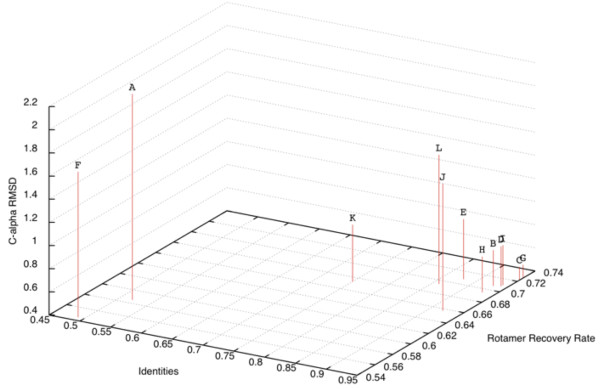
**Rotamer recovery rate, sequence identity, and C-alpha RMSD for model-crystal structure pairs.** The amino acid sequences for each crystal structure used as templates for the HASP server were modeled using the HASP server and the resultant models compared to the crystal structure. The identities are the number of identical amino acids between the query, template pair. The rotamer recovery rate is the ratio of residues with correctly modeled side-chains to all residues. The C-alpha RMSDs were calculated between the template structure and crystal structure of the sequence being modeled over all C-alpha atoms included in the model. Rotamer recovery *versus* identities can be fit by linear regression to the equation, rotamer recovery rate = 0.32 * identities + 0.41, with an R^2^ of 0.88. C-alpha RMSD *versus* rotamer recovery can be fit by linear regression to the equation, RMSD = −2.52 * identities + 3.16, with an R^2^ of 0.62. The PDB codes of the modeled sequence and template pairs are: A. 1JSD,3HTT; B. 1JSM,3GBM; C. 1MQM,2VIU; D. 1RV0,3GBN; E. 1RVX,3GBN; F. 1TI8,2VIU; G. 2VIU,1MQM; H. 3GBM,1JSM; I. 3GBN,1RV0; J. 3HTT,3GBN; K. 3KU3,3GBM; L. 3LZG,3GBN.

To derive semi-quantitative estimates of the quality that can be expected for modeling a given HA sequence, the sequences of the twelve HA crystal structures used as templates were modeled using the server, omitting their structures from the possible templates, and the models were compared to the crystal structures by various criteria (Figure 1, Table 2). The recovery rate of side chain conformations between the HASP-generated model and the crystal structure were calculated using the ROSETTA3 scientific benchmarking functions (Leaver-Fay A, O’Meara MJ, *et al.*; unpublished). The C-alpha RMSD between the template structure and the crystal structure were calculated. The rotamer recovery rate and C-alpha RMSD were evaluated as a function of sequence identity between the query sequences and sequences of the templates, the only information a user of the HASP server would have *a priori*. These relations could be fit by linear regression such that the rotamer recovery rate can be approximated as 0.32 * identities + 0.41, with an R^2^ of 0.88, and the C-alpha RMSD can be approximated as −2.52 * identities + 3.16, with an R^2^ of 0.62 (Figure 1). Given the limited dataset (n = 12), these equations are intended only to serve as an estimate of the quality of a model that can be expected for an HA query-template pair with a given sequence identity.

**Table 2:** Summary of rotamer recovery for template sequences modeled using the HASP server

**Amino acid**	**Total**	**Rotamers recovered**	**Recovery rate ± std**^**a**^
**D**	295	147	0.50 ± 0.11
**E**	379	152	0.40 ± 0.13
**F**	207	185	0.89 ± 0.11
**H**	154	116	0.75 ± 0.12
**I**	344	210	0.62 ± 0.10
**K**	355	124	0.35 ± 0.13
**L**	446	333	0.75 ± 0.08
**M**	96	35	0.37 ± 0.18
**N**	491	223	0.45 ± 0.11
**Q**	193	77	0.41 ± 0.12
**R**	233	86	0.37 ± 0.11
**S**	408	281	0.69 ± 0.14
**T**	398	293	0.73 ± 0.11
**V**	320	270	0.84 ± 0.07
**W**	109	105	0.96 ± 0.09
**Y**	244	231	0.95 ± 0.06

^a^The standard deviation (std) of the recovery rate was calculated over the set of individual recovery rates for each template sequence modeled (n = 12).

On a finer scale, the rotamer recovery rate was evaluated as a function of amino acid type (Table 2). Rotamer recovery rates were roughly proportional to hydrophobicity and the number of dihedrals for a given amino acid type. Large hydrophobic amino acids with fewer dihedrals (F, W, Y) were recovered at rates of approximately >90%. Amino acids with many dihedrals (K, R, M) were recovered at the lowest rates, 35-37%, followed by hydrophilic amino acids with fewer dihedrals (D, E, N, Q) with rates between 40-50%.

### Implementation

HASP is a web application written in Java 1.6, and is designed with standard client/server architecture. The meta-information from GenBank [12] and the HA models are stored in a MySQL 5.0 relational database. The server side utilizes Hibernate Search and an Apache Lucene text engine library to communicate with the MySQL database and perform keyword search. The Google Web Toolkit 2.3 (GWT) was used to render the client side interface. The viewer utilizes Google Chart Tools for geographical search and map visualization, and Jmol Applet 12.0 for structure visualization with JavaScript Native Interface bridges handling communication between GWT and Jmol. Multiple sequence alignments are generated dynamically using MAFFT 6 [13]. The Google Charts Tools Intensity Map is used to graphically show the number of strains collected in a given geographical area.

## Utility and discussion

### Main features of the HASP server

The HASP server interface has two primary components, the *Search* tab (buttons or tabs are identified hereafter in italics) to identify HA sequences of interest and the *Viewer* tab to display both the sequences and structures of the selected HA proteins. In the *Search* tab, HA sequences of interest can be selected from the database based on the H/N subtype of the strain, the geographical location of strain collection, by keyword, and other strain features.

To start, a user chooses the parameters of the query in the *Search* tab. By clicking *Toggle Map Viewer*, results can be narrowed by geographical location of strain collection through a color-coded world map; countries labeled in dark and light shades of green have the highest and lowest number of cases, respectively. Clicking *Go* updates the results, and after toggling out of the map viewer, a list of HA sequences is displayed. Information provided for each sequence includes the EMBL ID, Subtype, Strain, Year, Location (City, State/Country), and Species (host).

Sequences of interest are selected by checking the box in the right-hand column of the search results. In the *Viewer* tab, up to two of these models can be displayed simultaneously within the interface using the built-in molecular viewer. Residues can be easily identified within the model through interactive alignment functionality. In the viewer, preset views of the models may be selected through buttons (*Hydrogens On*, *All Atoms On*, *Single Chain*) with options for displaying models as cartoons, or with or without labels. For further analysis, a drop-down menu allows the user to export sequences or structures in FASTA or PDB format, respectively.

### Discussion of HASP server use through a case study

In the World Health Organization’s Human Animal Interface online database for H5N1 Avian Influenza in Humans, it is reported that there were three deaths from H5N1 in Vietnam in 2003 and twenty deaths in 2004 [14]. The differences between a pair of HA proteins sequenced from infected humans and chickens in Vietnam in 2003 and 2004 are explored below with the HASP server as a case study.

**Figure 2:**
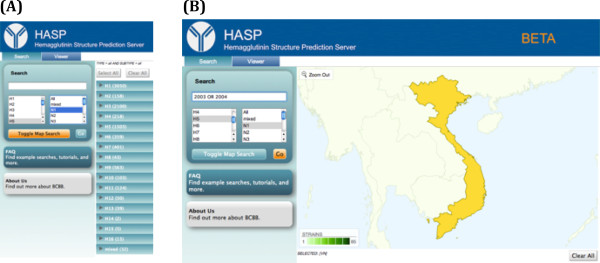
**Searching for hemagglutinin sequences.** (**A**) Opening view in the Search tab. The *H5* and *N1* subtypes have been selected and the *Toggle Map Search* button is clicked. (**B**) The map selection viewer. Vietnam has been selected from the map and “2003 OR 2004” has been added to the search box. The *Go* button is clicked.

**Figure 3:**
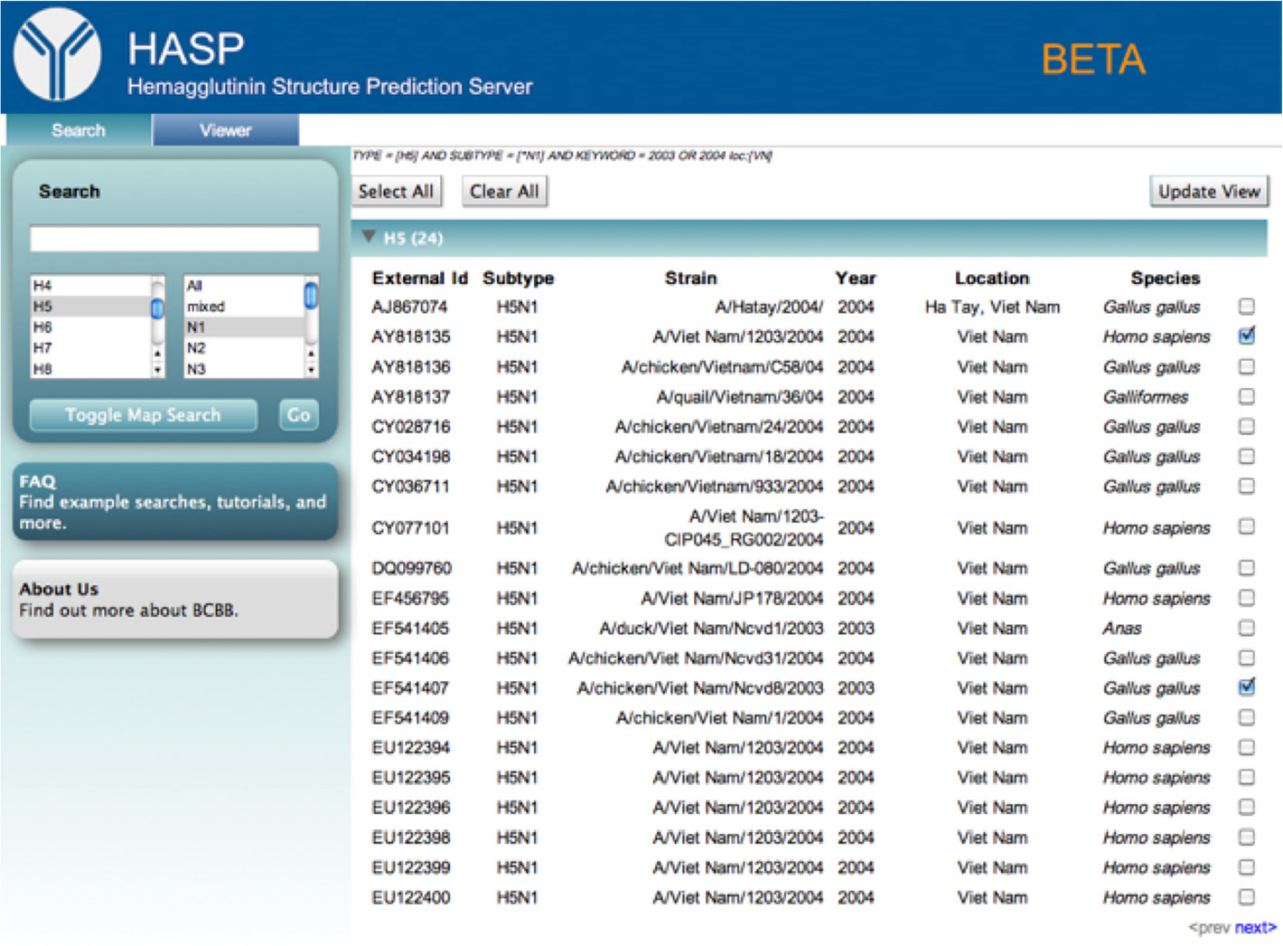
**Search results.** A/Viet Nam/1203/2004 (AY818135) and A/chicken/Viet Nam/Ncvd8/2003 (EF541407) are selected and the *Update View* button is pressed. The full query is shown above the *Select All* button: TYPE = [H5] AND SUBTYPE = [*N1] AND KEYWORD = 2003 OR 2004 loc:[VN].

To narrow the search to HA sequences from H5N1 strains, *H5* and *N1* are selected from the subtype menu below the *Search* box (Figure 2A). To refine the search to H5N1 strains by country, Vietnam is selected on the map. The search term, “2003 OR 2004”, is added to the search box to restrict the search to strains sampled in those years (Figure 2B). Clicking the *Go* button updates the query, and toggling out of the map viewer displays the results page, where twenty-four H5 sequences are found for the H5N1 subtype in Vietnam between 2003–2004 (Figure 3). To compare the HA sequences sequenced from human and chicken hosts, boxes are checked next to “A/Viet Nam/1203/2004 (AY818135) (human hosts)” and “A/chicken/Viet Nam/Ncvd8/2003 (EF541407) (chicken hosts),” Clicking *Update View* displays the two sequences in the *Viewer* tab.

**Figure 4:**
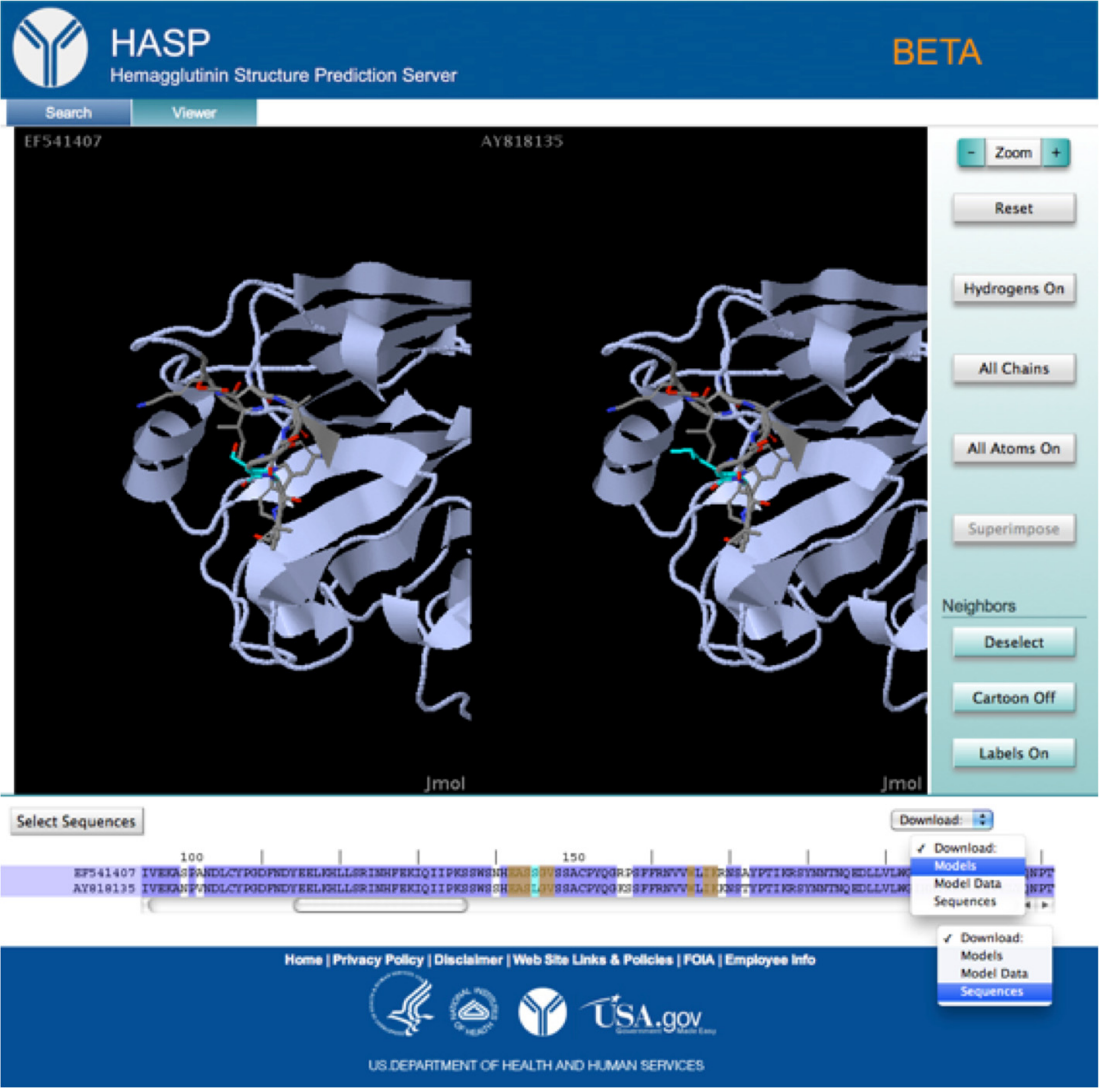
**The *Viewer *tab.** Both sequences in the alignment are selected, activating the side-by-side view mode. The *Single Chain* button has been pressed to clarify the view. Position 145 is selected, centering the H5 models in the viewer and highlighting that position in cyan and its neighboring positions in yellow. The residue and its neighbors are displayed as sticks on top of the ribbon diagram. The *Download* button has been pressed, showing the drop-down menu in which *Models* and *Sequences* have been selected for download.

In the viewer, a model is displayed for the selected 2003-chicken H5. To compare the two models, the identifiers for both sequences are selected at the left of the sequence alignment, highlighting them in purple and instantiating a side-by-side viewer (Figure 4). In order to clarify the view, the *Single Chain* button is clicked, which displays only one chain of each trimer. Scrolling through the alignment, a non-conservative substitution at position 145 adjacent the receptor-binding domain is observed. At this position, there is a serine in the chicken host H5 and a leucine in the human host H5. Clicking on this residue in the sequence highlights in cyan the corresponding residue in each model with neighboring residues highlighted in yellow in the alignment. The side-by-side view of each model is also centered on the residue of interest and it and its neighbors are rendered as sticks on top of the ribbon diagram. From the viewer, the leucine appears exposed in the human host H5 (Figure 4). To investigate this observation further through additional modeling and sequence analysis, the models and sequences are downloaded by pressing the *Download* button, and selecting *Models* and *Sequences*, respectively, from the drop down list.

## Conclusions

Because HA regulates IAV entry into the cell [15] and strongly impacts both pathogenicity and interspecies transmission [16,17], new insights into HA structure and function are critical contributions to the study of influenza A. The HASP server was designed to enable researchers to visualize their HA sequences of interest in the three-dimensional context of the most related HA crystal structure. It employs computationally fast protocols for model generation, database navigation, and visualization appropriate for the large number of HA sequences and scalable with increasing sequence data. These protocols will be used in planned implementations of the HASP server to allow for models to be generated in real-time for user submitted sequences. The HASP server makes structural information on HA sequences quickly and easily accessible to all researchers, providing a valuable aid for interpreting data and generating new hypotheses. For example, structural information derived from the HASP pipeline was recently utilized in a study of a HA variant from the 1918 influenza pandemic [18] that resulted in nearly 50 million deaths worldwide [19]. In that study, the models generated were used in docking studies with receptor analogs to assess potential changes in receptor binding specificity resulting from point mutations [18].

The primary aim of the HASP server is to provide a tool for viewing and comparing amino acid changes in HA subtypes at all levels of protein structure, from primary to quaternary, giving a complete and integrated view of those changes to facilitate understanding. Understanding these changes, which determine the efficiency of transmission, pathology, and ecology of these viruses, is of critical and vital importance to global public health.

### Availability and requirements

The HASP server is available free of charge as a web application at: http://exon.niaid.nih.gov/HASP.html

## Abbreviations

HASP: Hemagglutinin Structure Prediction (server); HA: Hemagglutinin; NA: Neuraminidase; RMSD: Root Mean Square Deviation; hsp: High-scoring segment pair; IAV: Influenza A virus; PDB: Protein Data Bank; GWT: Google Web Toolkit.

## Competing interests

The authors declare that they have no competing interests.

## Authors’ contributions

XIA, EJD, JKT, and DEH conceived the HASP server. XIA designed the HASP server, created the modeling pipeline, performed the model quality assessment, and authored the manuscript. JD led the development and implementation of the HASP server. VG assisted JD with the development and implementation of the HASP server. All authors contributed to and edited the manuscript, and approved the final version.
